# 470. Relapsing COVID-19 Pneumonia in Patients Receiving Rituximab Therapy

**DOI:** 10.1093/ofid/ofab466.669

**Published:** 2021-12-04

**Authors:** Jolie Gallagher, Kevin H Hall, Benjamin Albrecht

**Affiliations:** 1 Emory University Hospital, Atlanta, Georgia; 2 Winship Cancer Institute of Emory Healthcare, Atlanta, Georgia; 3 Emory Healthcare, Atlanta, Georgia

## Abstract

**Background:**

Rituximab is a monoclonal antibody against the CD20 antigen on B-lymphocytes leading to B-cell death and depletion. Patients who receive rituximab and are infected with the novel severe acute respiratory syndrome coronavirus-2 (SARS-CoV2) causing coronavirus disease (COVID-19) may have increased difficultly clearing the virus and be at risk for persistent disease. While the limited literature available is mixed regarding the severity of COVID-19 in patients receiving rituximab, there is minimal literature regarding persistent and relapsing COVID-19 in this patient population. This is a case series of patients with persistent COVID-19 who previously received rituximab.

**Methods:**

This is a retrospective review of 5 patients admitted between 1/1/2021 and 5/1/2021 to our institution with confirmed COVID-19 and receipt of rituximab for any indication within the previous 12 months. Information regarding hospital readmissions, time course of positive infection, medical management, disease severity, and discharge disposition were collected.

**Results:**

Five patients, median age of 46, currently or recently on rituximab therapy were admitted a median of 2 times due to persistent, severe COVID-19 (Table 1). Patients received their initial COVID-19 diagnosis a median of 34 days (8-102 days) since their last rituximab administration and had documented SARS-CoV-2 infection a median of 66 days (19-195 days; Figure 1). All 5 patients received remdesivir and corticosteroids over the course of their COVID-19 disease and 2 patients received convalescent plasma therapy 1 and 5 days prior to a positive SARS-CoV-2 antibody IgG.

Figure 1. Patient SARS-CoV-2 Infection Course

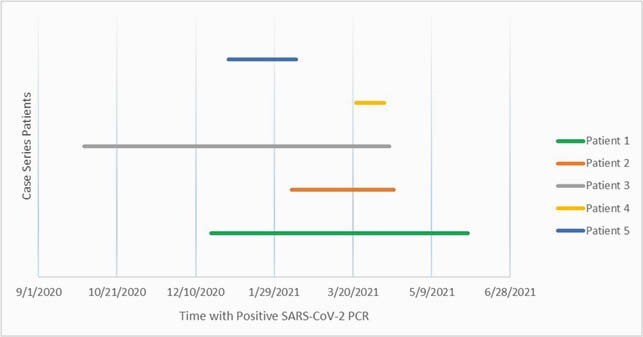

Table 1. Patient Clinical and Therapeutic Data

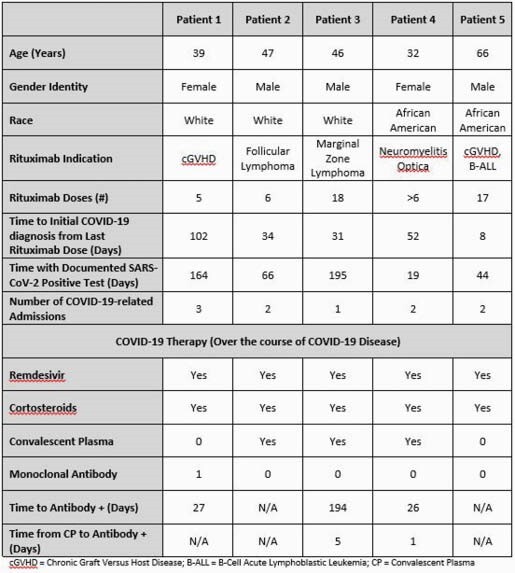

**Conclusion:**

Rituximab therapy may be associated with persistent or relapsing COVID-19 disease. Controlled investigations are necessary to evaluate the exact impact anti-CD20 agents have on the course of COVID-19 and whether convalescent plasma or other therapies can prevent relapsing disease.

**Disclosures:**

**All Authors**: No reported disclosures

